# Diagnosis of Swallowing Disorders: How We Interpret Pharyngeal Manometry

**DOI:** 10.1007/s11894-017-0552-2

**Published:** 2017-03-13

**Authors:** Charles Cock, Taher Omari

**Affiliations:** 1 Gastroenterology & Hepatology, Southern Adelaide Local Health Network, Adelaide, Australia; 2 Department of Gastroenterology & Hepatology, School of Medicine, Flinders University of South Australia, Flinders Medical Centre, Flinders Drive, Bedford Park, 5042 Australia; 3 Human Physiology, Medical Science and Technology, Flinders University of South Australia, Bedford Park, Australia

**Keywords:** Deglutition, Dysphagia, High-resolution manometry, Impedance, Pharynx, Pressure

## Abstract

**Purpose of review:**

We provide an overview of the clinical application of novel pharyngeal high-resolution impedance manometry (HRIM) with pressure flow analysis (PFA) in our hands with example cases.

**Recent findings:**

In our Centre, we base our interpretation of HRIM recordings upon a *qualitative* assessment of pressure-impedance waveforms during individual swallows, as well as a *quantitative* assessment of averaged PFA swallow function variables. We provide a description of two global swallowing efficacy measures, the swallow risk index (SRI), reflecting global swallowing dysfunction (higher SRI = greater aspiration risk) and the post-swallow impedance ratio (PSIR) detecting significant post-swallow bolus residue. We describe a further eight swallow function variables specific to the hypopharynx and upper esophageal sphincter (UES), assessing hypo-pharyngeal distension pressure, contractility, bolus presence and flow timing, and UES basal tone, relaxation, opening and contractility.

**Summary:**

Pharyngeal HRIM has now come of age, being applicable for routine clinical practice to assess the biomechanics of oropharyngeal swallowing dysfunction. In the future, it may guide treatment strategies and allow more objective longitudinal follow-up on clinical outcomes.

## Introduction

The purpose of pharyngeal deglutition is safe and effective propulsion of swallowed material (bolus) through the pharynx and upper esophageal sphincter (UES), attuned to the physical characteristics of the bolus. Hence, in order for pharyngeal deglutition to be safe and effective, oral-lingual preparatory and propulsive mechanisms, airway protective mechanisms, UES relaxation and opening mechanisms and pharyngo-esophageal propulsive mechanisms need to be adaptable to boluses of differing volume, consistency and rheological characteristics [1•, 2•, 3, 4••, 5]. Failure of these mechanisms leads to abnormal bolus transport, causing aspiration due to miss-direction into the lungs [6••] or the retention of pharyngeal residue, which poses an increased aspiration risk for subsequent swallowing [7•]. A critical component of normal pharyngeal deglutition is adequate upper UES relaxation and sufficient UES opening. Pharyngeal manometry has been used to assess UES relaxation, pharyngeal propulsion and the related intra-bolus pressure, as a marker of UES restriction [8, 9].

Clinicians have largely ignored pharyngeal manometry. Traditionally, pharyngeal manometry is performed in concert with radiological imaging [10, 11], requiring specialised, often purpose built, video-manometry equipment. The emergence of high-resolution solid-state impedance manometry (HRIM) catheters, combined with pressure topography and pressure-flow analytics, has changed this landscape. The video-HRIM equipment, whilst expensive, is now commercially available throughout the world. The reliable recordings obtained are more easily interpretable than ever before, allowing clinicians to gain the knowledge to implement HRIM into routine clinical practice.

In this paper, we describe how we analyse pharyngeal HRIM recordings in our Centre and how we use this information to guide diagnosis and treatment of dysphagia symptoms. We hope that the information contained here will help to remedy any misconceptions regarding the potential value of pharyngeal HRIM.

## What Does High-Resolution Manometry With Impedance Measurement Add?

HRIM adds a wealth of information on the biomechanics of swallowing dysfunction, which serves to inform both diagnosis and treatment strategies. Through the addition of impedance to the manometry, the “pressure-flow structure” of the swallow can be accessed without the need for radiology and is therefore potentially applicable for use in settings where access to a radiology suite is limited, including rural or remote settings [12–14].

**Fig. 1:**
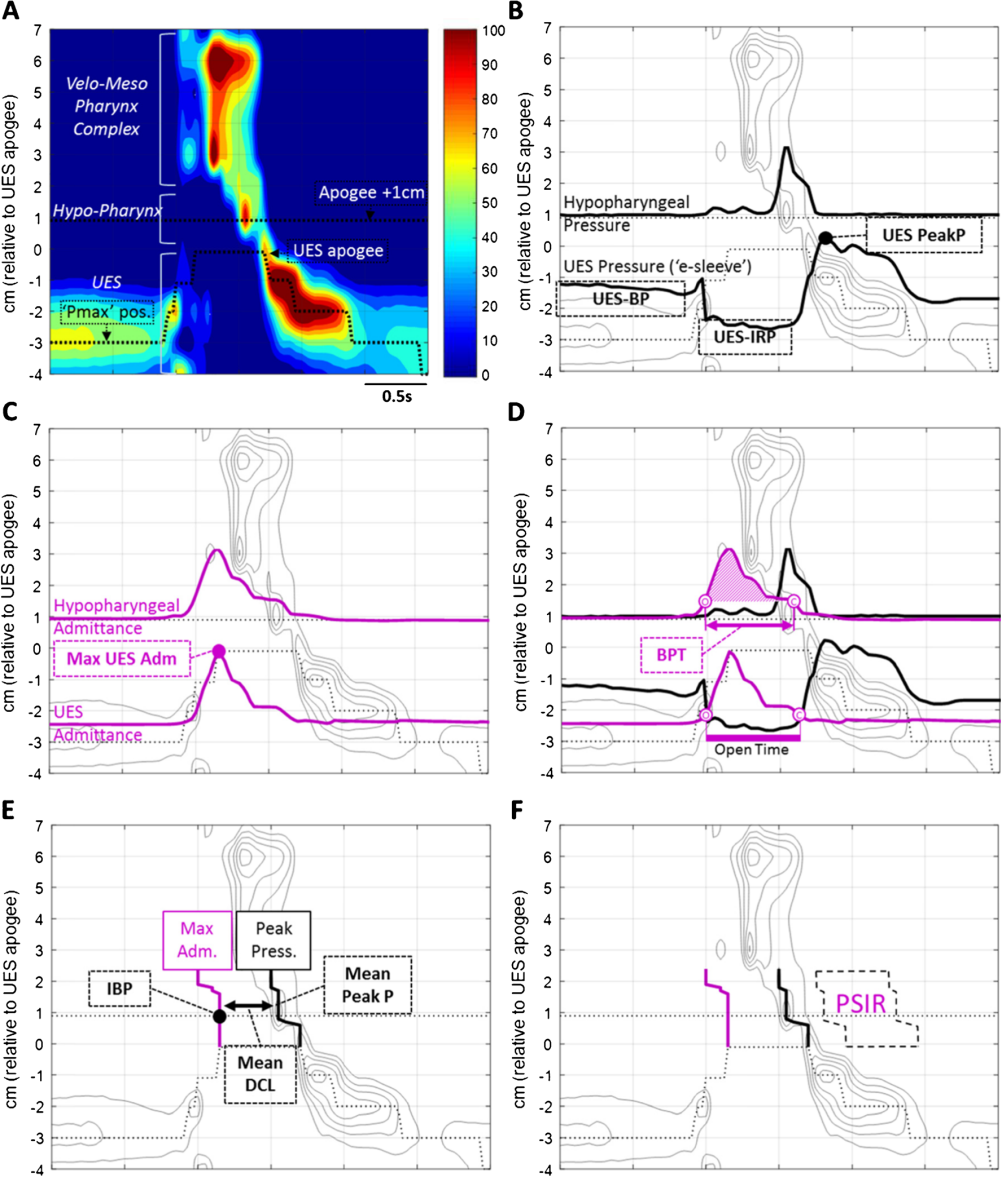
*Swallow function variables based on HRIM recordings*. **a** High-resolution colour pressure topography plot of a 10-ml saline bolus swallow recorded in a healthy subject. *Scale right* shows the range of pressure (*blue* indicates lowest pressure, *red* indicates highest pressures). Pressure patterns allow the pharyngeal chamber to be separated into three regions; velo-/meso-pharynx complex, hypopharynx and upper esophageal sphincter (UES). The *dotted line within the UES region* shows the axial location of maximum UES pressure during the swallow (Pmax position) tracking a ∼3-cm superior movement of UES high pressure zone from resting to apogee position (0 cm). The *dotted line within the hypopharynx* indicates the position 1 cm proximal to the UES apogee (+1 cm) which is the standard location we use to define hypo-pharyngeal pressure and admittance variables (see below). **b** The same pressure topography plot with colour removed showing isobaric contours from 40 to 120 mmHg. The *upper black line* shows the pressure waveform recorded at the hypo-pharyngeal position during the swallow (apogee +1 cm) and the *lower black line* shows UES pressure waveform constructed from pressures recorded at the Pmax position over time. From these data, the mean pre-deglutitive UES basal pressure (*UES-BP*), UES integrated relaxation pressure (*UES-IRP*) and post-deglutitive UES peak pressure (*UES-PeakP*) can be determined. **c** The same pressure topography plot with colour removed showing isobaric contours from 40 to 120 mmHg. The *upper purple line* shows the admittance waveform recorded at the hypo-pharyngeal position during the swallow (apogee +1 cm) and the *lower purple line* shows UES admittance waveform constructed from impedance recorded at the Pmax position over time. Note: Admittance (in Siemens, S) is the *inverse product* of impedance (*S* = 1/Ω); therefore, the admittance *rises* with bolus distension of the hypopharynx and UES and the maximum admittance within the UES (*Max UES Adm*) is indicative of maximum cross-sectional area of the lumen. **d** The same plot as in C, however now showing how the UES admittance and pressure waveforms can be used together to define the onset of UES opening (*O*), based on the admittance upstroke within the UES, and UES closure (*C*), based on the pressure upstroke within the UES. For estimation of hypo-pharyngeal bolus presence time (*BPT*), the UES admittance level recorded at the time of UES closure (*C*) is applied as a threshold to the pharyngeal admittance recording; hence, the period that the pharyngeal admittance exceeds this threshold defines bolus presence time (period from O to C marked on the hypo-pharyngeal admittance waveform). **e** The same pressure topography plot with colour removed. However, in this figure the *lines* indicate the time of maximum admittance (*Max Adm*.) and peak pressure generation (*Peak Press*.) along the hypo-pharyngeal region, indicating the time of maximum bolus distension and maximum contraction of the hypopharynx during the swallow. Hypo-pharyngeal intra-bolus distension pressure (IBP) is defined by the pressure recorded at maximum distension, 1 cm proximal of the UES apogee. Hypo-pharyngeal mean peak pressures (*mean Peak P*) define maximum contractility of the pharyngeal constrictors. The average latency from maximum distension to peak contraction (*DCL*) defines the timing of flow relative to contraction. **f** The same pressure topography plot with colour removed showing the post-deglutitive hypo-pharyngeal region used for calculation of PSIR

High-resolution manometry with impedance provides a visual depiction of pressure flow during pharyngeal deglutition, assessable qualitatively by those with expertise. Objective swallow function variables can also be rapidly derived across several swallows by non-experts using software. Together, this information can detect abnormalities that may explain dysphagia symptoms. Several swallow function variables, we believe, are relevant to understanding the health of the swallowing mechanism using HRIM. These we describe below (see also Fig. 1) [12–14, 15•, 16••, 17, 18•, 19, 20, 21•, 22•, 23••, 24••].

### Key Swallow Function Variables for Dysphagia Assessment

Characterising UES relaxation and opening is important for the assessment of swallowing disorders. UES relaxation/opening failure can occur due to structural pathology, problems with integrative neuro-sensory function and/or failure of neuro-muscular mechanisms. Relaxation and opening of the UES is a complex process, modulated via sensory inputs, changing the duration of relaxation and the extent of opening in relation to the bolus swallowed. Larger boluses travel at a faster flow rate through the pharyngo-esophageal segment, and normal swallowing allows this to occur without substantially increasing the flow resistance [1•, 2•, 3–5]. Hence, by changing the volume and consistency of the boluses swallowed, it may be possible to reveal structural pathology or sensory misregulation of swallowing.

At rest the UES can be identified by the presence of a high pressure zone generated by cricopharyngeus (CP) muscle contraction. During swallowing, the CP muscle deactivates, remains inactive whilst the UES lumen opens then closes, then the CP re-activates. The suprahyoid muscles, mechanically coupled to the UES, and the distension forces generated during bolus passage govern luminal opening/closure during CP inactivity. Whilst manometry alone cannot easily elucidate UES opening and closure, radiology, or its non-radiological surrogate, intraluminal impedance denoting bolus presence and flow, can [2•].

We routinely assess UES tonic contractility using pre-deglutitive basal pressure (UES-BP, Fig. 1b) and extent of UES relaxation using the UES integrated relaxation pressure (UES-IRP, Fig. 1b) [16••, 25, 26•]. At this time, there are limited published data available from patients with UES obstruction. However, UES IRP values have been shown to increase with age [16••, 26•] and are elevated in subjects with motor neurone disease [16••], but interestingly not consistently in subjects with radiological cricopharyngeal bars [16••], showing that this pathology is not always obstructive in nature.

We use impedancometric measurements to determine information on pharyngeal and UES opening. The *nadir impedance* value [17] during bolus swallowing, or its inverse, *maximum admittance* (used henceforth), can be used to infer the maximum extent of luminal opening during bolus flow. When measured within the UES region, the maximum UES admittance (Max UES Adm, Fig. 1c) is reduced in dysphagic patients and therefore appears to be an excellent non-specific measure for UES dysfunction [16••].

The hypo-pharyngeal maximum admittance, corresponding to maximum distension of the hypopharynx, objectively defines a time point for determining intra-bolus pressure during maximum bolus distension (IBP, Fig. 1e). IBP is the mechanical consequence of lingual and pharyngeal propulsive forces and the diameter of the lumen. Per volume swallowed, an abnormally high IBP can be a marker of flow restriction due to structural pathology [18•, 19, 20] or, alternatively, a high IBP can be a marker of sensory misregulation of the swallow leading to failure to accommodate for bolus size and the bolus transiting the pharyngeal chamber more rapidly [21•].

An important caveat when using IBP diagnostically is that IBP can only discriminate a pathological UES restriction of bolus flow when the pharynx has sufficient contractility to both propel the swallowed bolus distally and seal the lumen proximal of the bolus domain, preventing retrograde bolus escape [9, 19]. We use mean hypo-pharyngeal peak pressure (PeakP, Fig. 1e) and UES peak pressure (UES PeakP, Fig. 1b) to define the strength of contractility. These pressures are influenced by several factors. The strength and sequencing of neural activation and muscle fibre density of the swallowing muscles are most important. However, other passive factors, such as luminal diameter and pharyngeal wall thickness, are also important as they determine the degree of muscle shortening required in order to bring the lumen to a point of closure (i.e. influencing the length-tension properties of the swallowing muscles). Pharyngeal pressures are reduced in neuromuscular diseases such as motor neuron disease [16••] and other diseases affecting the musculature directly such as myopathies or myositis [20]. Interestingly, subjects with cricopharyngeal bars [16••] also have reduced pharyngeal pressures, implying this phenomenon may be a manifestation of pharyngeal neuromyopathy. Overall, abnormalities of pharyngeal contraction are rare and weakness should prompt a determined search for an underlying neuromuscular pathology. It is also important to keep in mind the important function of suprahyoid muscles in opening the UES, so that a reduction in UES opening (seen as a *low* UES Max Adm) can occur in concert with pharyngeal weakness, because of muscle weakness more globally.

Finally, in addition to guiding IBP measurement, the admittance profile of the hypopharynx provides an estimate of the bolus presence time (BPT, Fig. 1d) and the latency period from distension (maximum admittance) to contraction (DCL, Fig. 1e). Mistiming of swallow coordination is likewise an important determinant of pre-swallow aspiration [22•]. A short BPT or DCL could indicate perturbation of sensory afferent mechanisms leading to an inability to modulate the swallow to accommodate different volumes. Poor-oral bolus containment (failure of lingual propulsion) results in a short DCL (because the major forces driving propulsion switch from predominantly lingual to being predominantly pharyngeal; refer to descriptions of the phases of bolus flow in the “Qualitative Assessment of Swallow Recordings” section below). Whilst a long BPT may suggest pre-swallow bolus presence and/or post-swallow residue [22•].

### Global Swallow Function Variables

**Fig. 2:**
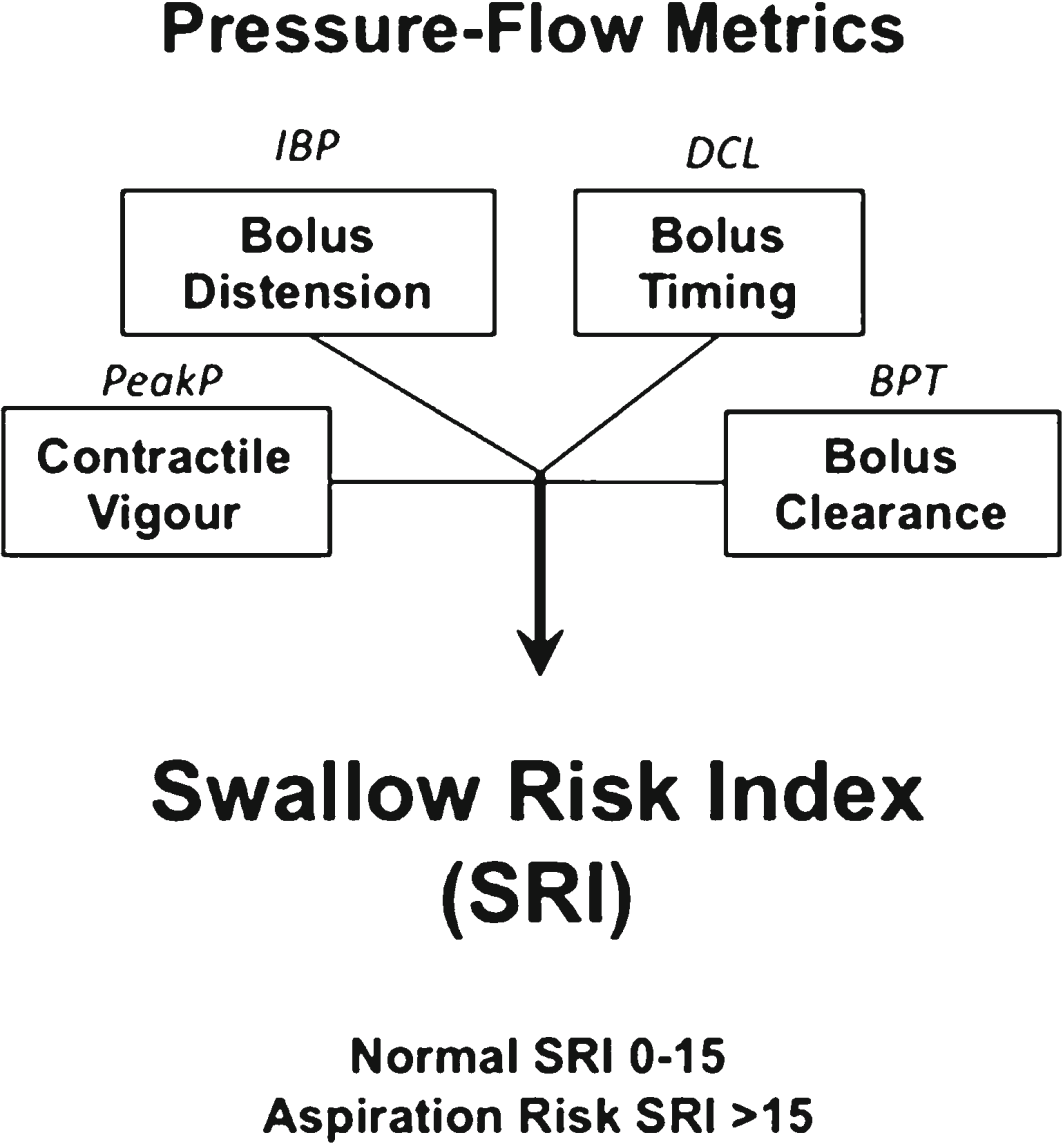
Key swallow function variables combined to derive the swallow risk index

We use two measures to determine global pharyngo-esophageal swallowing dysfunction; namely the swallow risk index (SRI) to estimate aspiration risk [23••] and post-swallow impedance ratio (PSIR) to estimate post-swallow residue [24••]. The SRI (Fig. 2) formula [23••] combines four hypo-pharyngeal measures to derive a single value representative of global swallowing dysfunction and aspiration risk. Post-swallow residue is an important determinant of aspiration on subsequent swallowing [7•]. Impedance values are lower (admittance higher) when the pharyngeal chamber contains bolus residual [24••, 27]. The PSIR relates impedance during swallowing to the impedance after pharyngeal contraction to derive a single value correlating the degree of post-swallow residual [24••] (Fig. 1f).

### Qualitative Assessment of Swallow Recordings

As pharyngeal swallowing and UES relaxation occurs rapidly, it can be challenging to assess the recordings without image manipulation. We find it most helpful to magnify the recording so that a 3–5-s interval fills the entire screen

**Fig. 3:**
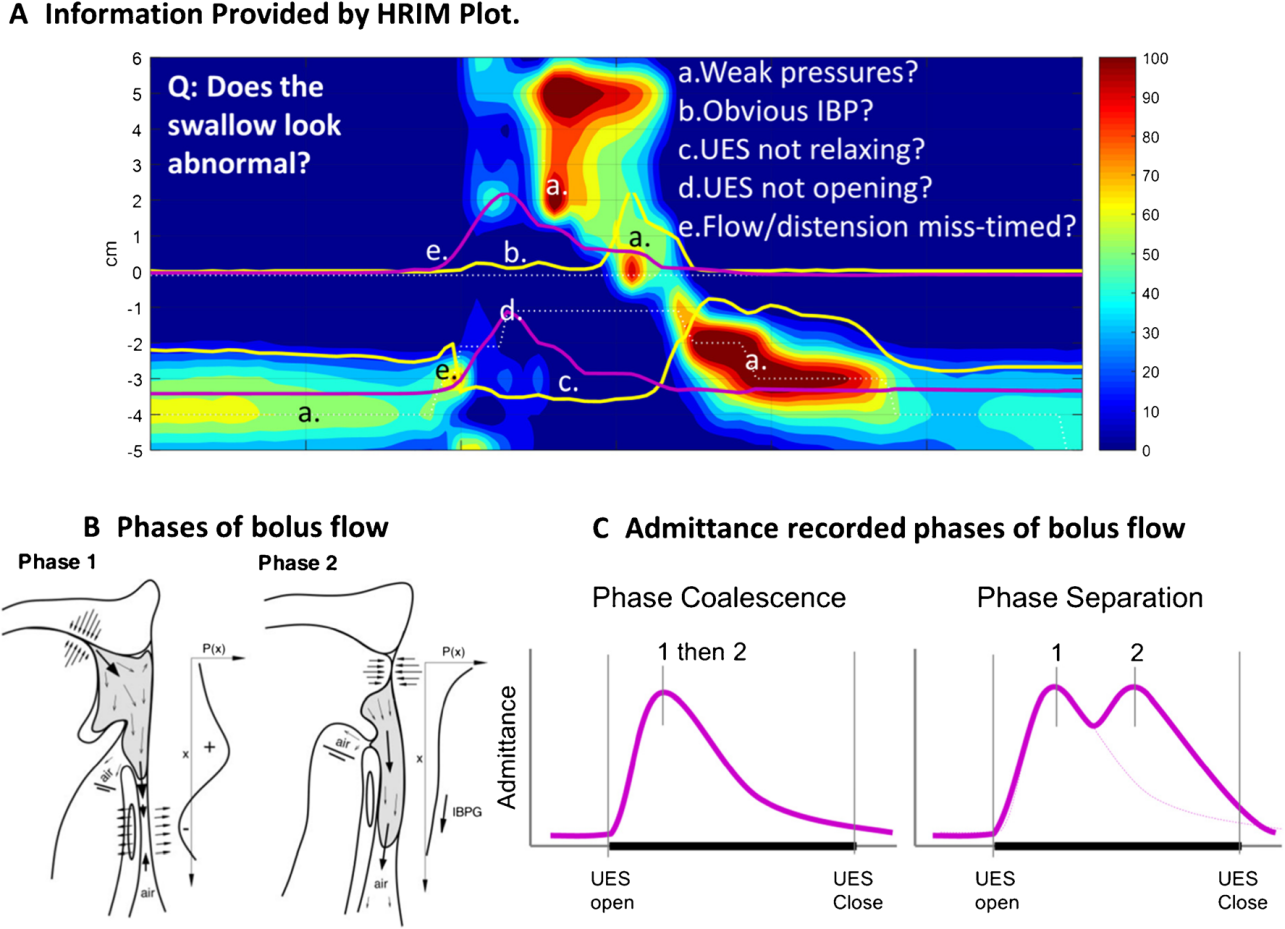
Qualitative assessment of a swallow recorded by HRIM. **a** Plot format ideal for qualitatively assessing pressure-admittance patterns during swallows. A high-resolution colour pressure topography plot of a 10-ml saline bolus swallow recorded in a healthy subject is shown (same as Fig. 1) with superimposed pressure waveforms (*yellow lines*) and admittance waveforms (*purple lines*) for the hypopharynx (*upper*) and UES (*lower*). Equivalent patient case examples are provided in Figs. 4, 5, 6, 7 and 8. **b** Illustration from Pal et al. [10] (reproduced with permission) showing the two phases of bolus motion during normal swallowing. Phase 1 is tongue-induced bolus acceleration. Phase 2 is bolus motion at a constant speed driven by the pharyngeal stripping wave. **c** How the hypo-pharyngeal admittance waveform potentially allows detection of aberrant timing of bolus propulsion. The *figure left* shows the single peaked waveform typical of normal circumstances of optimally timed UES opening to the propelled bolus, i.e. the lingual and pharyngeal phases are coalescent. The *figure right* shows a double peak waveform, exemplary of either mistiming of the phases or flow resistance specific to pharyngeal phase

As shown in Fig. 3a, each swallow can be qualitatively assessed for weak pressures at the velopharynx and tongue base, hypopharynx (including discreet gaps in the pressure sequence) and at the UES. UES relaxation and mistiming of flow events can also be assessed. Specific note should be taken of simultaneous “pan-pharyngeal” intra-bolus pressurizations, which are never seen in health and indicative of distal obstruction in combination with non-lumen occlusive pharyngeal contraction.

Qualitative information is also contained in the admittance and pressure curves in the hypopharynx and at the UES. The hypo-pharyngeal admittance during pharyngeal deglutition can often show separate phases of bolus flow. As originally described by Pal et al. [8], there are *two phases of bolus motion* during normal swallowing (Fig. 3b). *Phase 1* is dominated by tongue-induced bolus acceleration (lingual propulsion). In normal circumstances, UES opening occurs as the bolus encounters the UES producing little of no resistance to bolus flow during phase 1. During *phase 2*, the bolus is driven through the open UES at a constant speed by the pharyngeal stripping wave (pharyngeal propulsion). The hypo-pharyngeal admittance waveform is indicative of this bolus motion (Fig. 3c). In health, the waveform usually appears as a *single peak*, with the initial rise of admittance from baseline corresponding to when lingually propelled bolus enters the hypopharynx (closure of velopharynx can also be seen to occur at this point) and the admittance peak corresponding to maximum luminal distension. A single peak suggests that lingual and pharyngeal propulsion phases are coalescent. Appearance of a *double peak*, meaning that the phases are separately discernible from each other, may suggest either the following: (i) phase miss-timing (e.g. phase 1 precedes UES opening) and/or (ii) that the rate of flow during one of the phases is being disproportionately slowed (e.g. flow resistance during phase 2).

We have provided some more information and examples of the above commentary in the “Example Patient Cases” section below.

## Utilising Changes in Volume and Viscosity

Sensory modulation of the pharyngeal swallow response occurs during swallowing of different volumes and viscosity [1•, 2•, 3–5]. This sensory modulation may be lost during disease processes that affect the sensory system, such as longstanding uncontrolled diabetes mellitus or medications acting on the sensory afferent pathways, e.g. opioids [21•, 28]. It has recently been shown that the pharyngo-UES system is optimally attuned to swallowing bolus of 10 ml volume [2•]. Increasing volume beyond 10–15 ml enables us to observe perturbations in sensory modulation, manifesting as increased intra-bolus pressures as the complete bolus is not cleared from the hypopharynx. It is best to employ this strategy during video-HRIM studies, where swallowing safety is directly observed.

## What Patients Do We Study With Pharyngeal HRIM?

Pharyngeal HRIM provides biomechanical swallowing information in patients with oropharyngeal dysphagia, i.e. those who present with dysphagia with coughing, choking, nasal regurgitation and/or neuromuscular and neurodegenerative pathologies commonly associated with oropharyngeal dysphagia. HRIM has great potential to guide therapeutic interventions such as swallowing exercises or UES dilatation, Botox injection or myotomy and monitor, in an objective way, such intervention or otherwise disease progression.

The decision to perform HRIM after initial modified barium swallow (MBS), during MBS (video-HRIM) or as a stand-alone test without MBS depends on the individual patient circumstances. Aspiration risk, complex and/or unilateral pathologies should be studied either during MBS or following an MBS establishing swallowing safety. Patients that are studied using HRIM alone include those in whom clinical assessment suggests a very low baseline risk of aspiration, where an MBS has been previously performed, where a repeat HRIM study is being performed to assess longitudinal change and/or to evaluate the impact of a therapeutic regimen or intervention. We believe that HRIM is very viable for non-radiologically assessing biomechanical changes. Our own test-retest reliability data in healthy young and old subjects suggests that timing and flow measurements in particular, including the SRI, are reproducible from week to week [15•].

## Performing the Study

Prior to performing the study, the patient is familiarised with the procedure, questionnaires completed and consent obtained. Pharyngeal manometry to assess physiological swallowing is performed with the patient sitting. Topical anaesthesia (lignocaine gel to the catheter and spray to the nares and throat) is judiciously applied, but if sensory testing is a priority, this step can be omitted. After 5 min, the manometry catheter is placed via the anaesthetised nostril. The subject is requested to take sips of water when the catheter is at approximately 15 cm depth to aid passage of the catheter through the UES and the catheter is then advanced to approximately 35–40 cm depth (usual depth for esophageal studies is approximately 50–55 cm). Pressure from velopharynx to proximal esophagus should be visible on the manometric tracing. Following insertion, a minimum of 5 min should be allowed for accommodation.

Boluses are orally administered via a 20-ml syringe by an assistant. In most patients with no concerns regarding risk of aspiration, we start with 5 ml liquid boluses. For impedancometric studies, both liquid and viscous boluses need to be conductive with standardized conductivity (0.9% normal saline or barium with salt solution during video-manometry). Cued volitional swallows are performed. Double swallows should be repeated if possible. After 5 ml liquids, we progress to 10 ml and, in some cases, 20 ml liquid and standardized viscous boluses (e.g. Sandhill Scientific standardized “EFT” viscous). If there is coughing or a “wet voice”, a more conservative protocol can be employed by reducing the number of repeat swallows to three and *not* increasing the volume beyond 10 ml. Following completion of this standard protocol additional swallows, swallow manoeuvres or a 100-150 ml water swallow test can be added according to clinical need (however evidence for the clinical value for these is currently lacking).

## Diagnostic Approach During Pharyngeal Manometry

Our approach attempts to identify abnormal biomechanics, which may possibly explain clinical symptoms/radiological findings, and to define the optimal therapeutic approach.

In the first instance, we review the recorded swallows qualitatively, looking for pressure-impedance patterns that suggest abnormality. We then turn to the mean values for swallow function variables to determine if any lie outside our laboratory system specific normative values (Table 1).

**Table 1:** Normative values for novel swallow function variables

Metric	Meaning when abnormal	Normative range (P 10–90%)	
Global dysfunction			
Swallow risk index SRI	Global swallowing dysfunction (>15)	5 ml L 10 ml L 5 ml V 10 ml V	0–11 0–10 0–10 0–8
Residue—post swallow impedance ratio PSIR	Post-swallow residue	5 ml L 10 ml L 5 ml V 10 ml V	143–370 139–339 204–424 174–393
UES dysfunction			
UES maximum admittance UES Max Adm (mS)	Reduced UES opening (non-specific for mechanism)	5 ml L 10 ml L 5 ml V 10 ml V	4.4–9.1 5.9–12.4 3.2–5.4 4.2–5.8
Hypo-pharyngeal intra-bolus pressure at 1 cm above UES IBP (mmHg)	Pharyngeal outflow resistance	5 ml L 10 ml L 5 ml V 10 ml V	−1–22 –1–28 –2–23 –2–21
UES 0.25 s integrated relaxation pressure UES IRP (mmHg)	UES restriction	5 ml L 10 ml L 5 ml V 10 ml V	–4–15 –3–15 –2–15 2–17
Weak contractility			
Mean pharyngeal peak pressure PeakP (mmHg)	Weak hypo-pharyngeal contractility	5 ml L 10 ml L 5 ml V 10 ml V	69–280 83–292 75–272 76–268
UES basal pressure UES Basal P (mmHg)	Reduced pre-deglutitive tone	5 ml L 10 ml L 5 ml V 10 ml V	29–145 28–145 22–119 29–127
UES post-deglutitive peak pressure UES Peak P (mmHg)	Reduced UES contractility	5 ml L 10 ml L 5 ml V 10 ml V	149–548 170–605 156–541 170–567
Mistiming			
UES bolus presence time BPT (s)	Early arrival of bolus due to poor oral control and/or post-swallow residual	5 ml L 10 ml L 5 ml V 10 ml V	0.50–0.98 0.54–0.92 0.42–0.62 0.43–0.68
Distention contraction latency DCL (ms)	Aberrant flow timing	5 ml L 10 ml L 5 ml V 10 ml V	317–598 396–650 278–464 329–512

Normative ranges for swallow function variables. Subjects (*n* = 50; aged 20–79 yrs) swallowed 5 × 5 and 10 ml thin liquid bolus (L; 0.9% saline) and moderately thick viscous bolus (V; Sandhill “EFT” viscous medium). Recordings were obtained with a 3.2-mm Unisensor Catheter using the MMS, “Solar GI”, system

When interpreting the swallow function results, we look for evidence of global pharyngeal swallowing dysfunction based on a *high* SRI, suggesting global aspiration risk and/or *high* PSIR, indicating post-swallow residue.

If the observed swallows are qualitatively aberrant or one or both of these global measures are abnormal, then we interrogate the remaining measures to ascertain which components of the swallowing mechanism may be contributing to this dysfunction. In general order of importance, these are as follows:

1. *low* UES Max Ad, indicating reduced UES opening.
2. *high* IBP and/or UES IRP, as evidence of abnormal flow resistance.
3. *long* BPT, indicating pre-swallow bolus presence and/or post-swallow residual.
4. *short* DCL as evidence for delayed flow due to swallow sensory-motor misregulation, when IBP is normal, or flow resistance, when IBP is abnormal.
5. *low* UES basal P, PeakP or UES Max P as evidence for weak pharyngeal/cricopharyngeal contractility.

Each component should be viewed in concert with other components of pharyngeal swallowing, but also interrelated with upstream (oral) and downstream (esophageal) swallowing. Some of the abovementioned components can be interpreted through pressure measurement only; however, interpretation of more complex biomechanics and functional consequences need the use of impedance manometry and analytic software algorithms [12–14, 15•, 16••, 17, 18•, 19, 20, 21•, 22•, 23••, 24••].

## Example Patient Cases

**Fig. 4:**
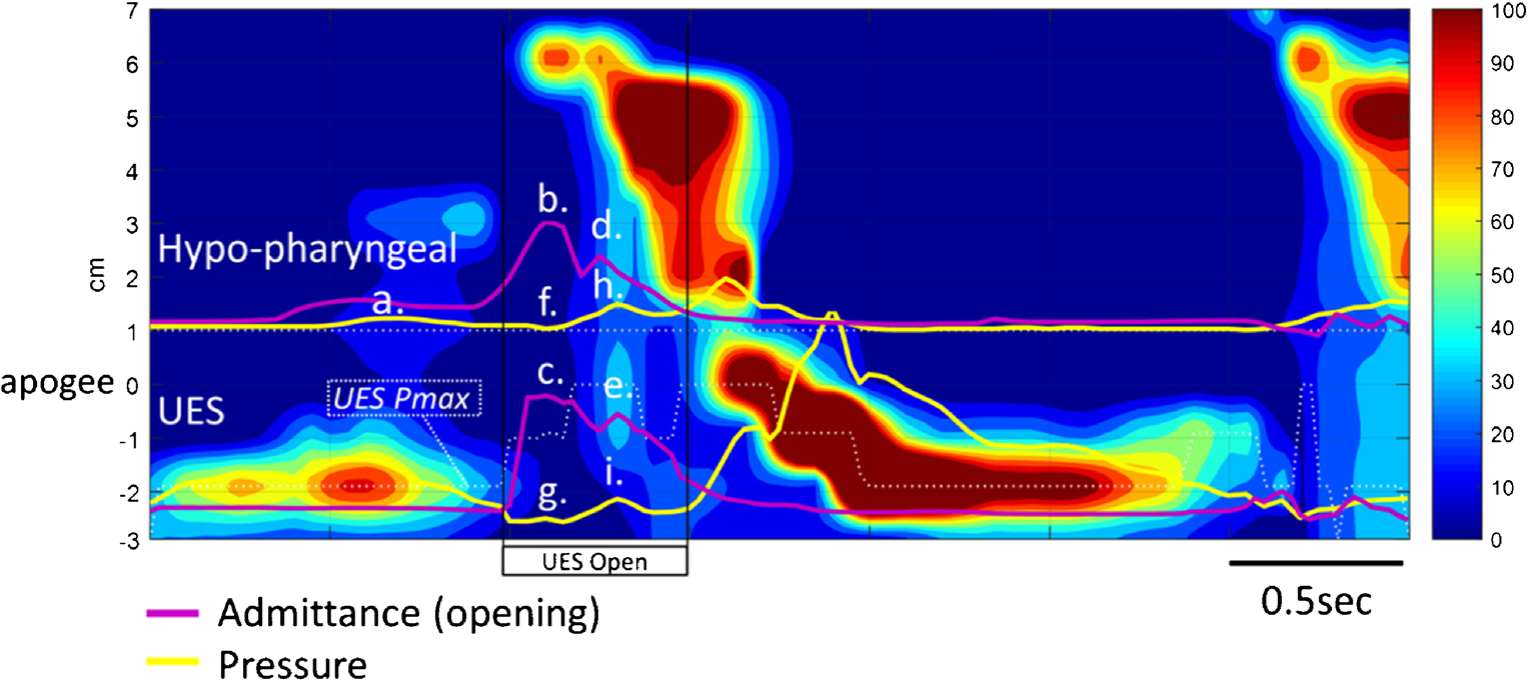
Example case 1 (C1 in Fig. 9) from a 64-year-old female patient 12 months following surgery and chemoradiotherapy for oropharyngeal cancer. Qualitatively, pharyngeal pressure topography suggests good pressure generation/contractility of the pharynx and UES, complete UES relaxation and evidence of pharyngeal intra-bolus pressure. Hypo-pharyngeal admittance shows evidence of pre-swallow bolus presence (higher level of admittance at *a*.). Both hypo-pharyngeal and UES admittance waveforms show a separation of lingual (*b*. and *c*.) and pharyngeal (*d*. and *e*.) propulsion. Note that the hypo-pharyngeal and UES pressures are low during lingual propulsion (*f*. and *g*.) but then increase in concert with admittance during pharyngeal propulsion (admittance increasing at *d*. and *e*.; pressure increasing at *h*. and *i*.). This pattern, when pressure and admittance increase together, is consistent with the lumen being passively distend due to forces generated above acting against a point of resistance. In this case the point of resistance is inferior to the UES because both the hypopharynx and UES are being passively distended by pharyngeal propulsion. Whilst an abnormal pattern, the average level of IBP (shown in Fig. 9—IBP for C1) was within normal ranges

**Fig. 5:**
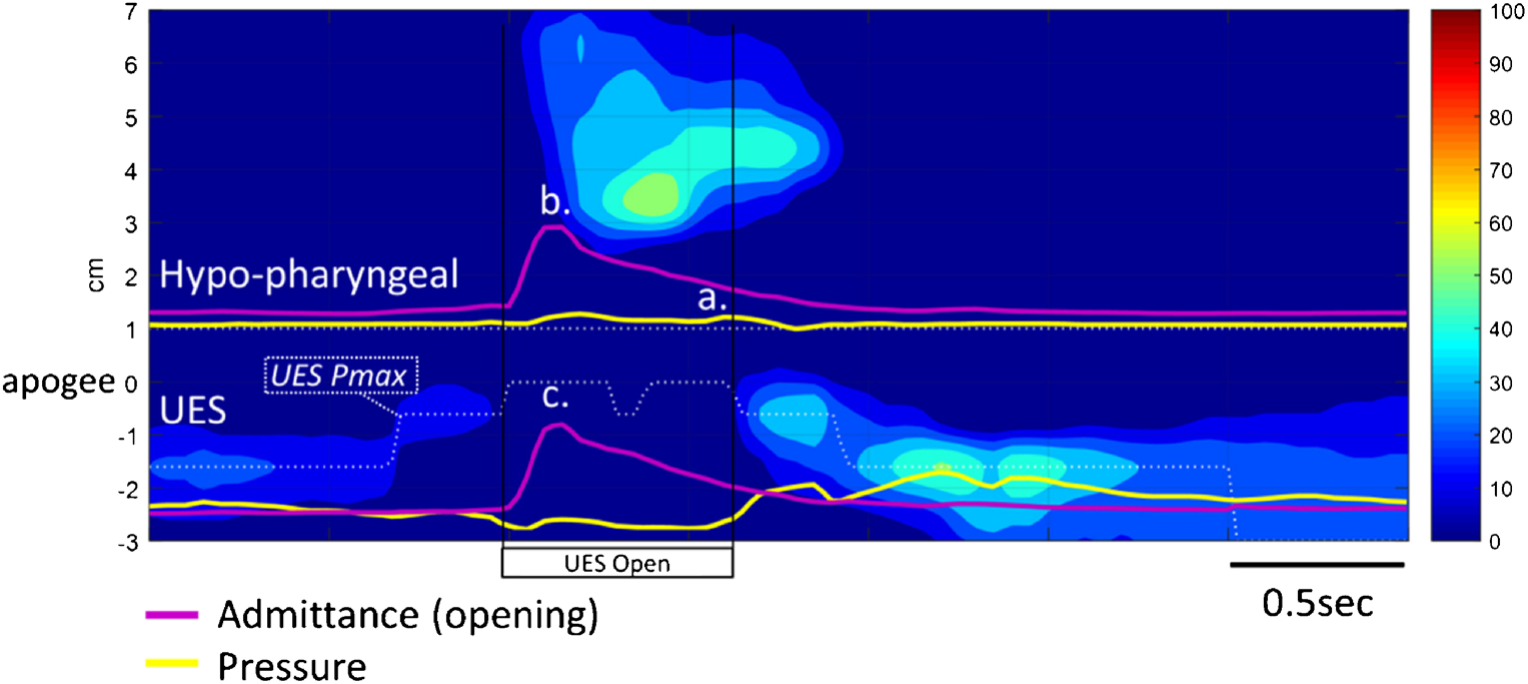
Example case 2 (C2 in Fig. 9) from a 64-year-old female patient with inclusion body myositis. Qualitatively, the pharyngeal pressure topography shows globally weak pressure generation/contractility of the pharynx and UES. Hypo-pharyngeal pressures are notably absent (*a*.). Hypo-pharyngeal and UES admittance waveform increases substantially from baseline and is single peaked consistent with adequate and appropriately timed lingual propulsion (*b*.) and UES opening (*c*.)

**Fig. 6:**
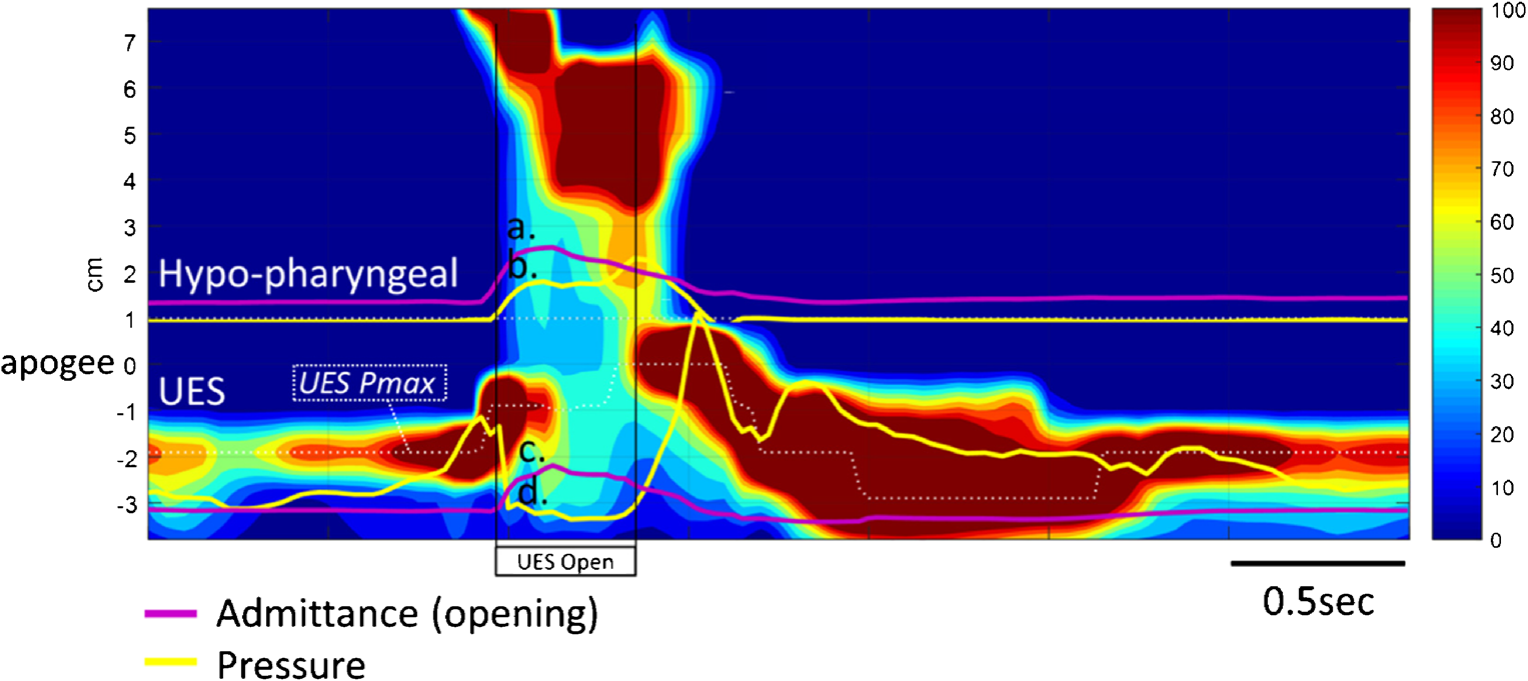
Example case 3 (C2 in Fig. 9) from a 68-year-old male patient with an obstructive pathology occluding the lumen at the level of UES. Qualitatively, the pharyngeal pressure topography suggests good pressure generation/contractility of the pharynx and UES, incomplete UES relaxation and elevated pharyngeal intra-bolus pressures. Hypo-pharyngeal admittance shows no evidence of pre-swallow bolus presence and no clear separation of the lingual and pharyngeal phases of propulsion. Note that the hypo-pharyngeal distension admittance (*a*.) and pressure (*b*.) rise together (passive distension). However, in contrast to the previous case 1 (Fig. 4), passive distension occurs immediately with the onset of lingual propulsion and is only present within the hypopharynx, not in the UES (compare admittance and pressure change at *a*. and *b*. with *c*. and *d*.). This pattern is consistent with flow resistance at the level of the UES rather than below

**Fig. 7:**
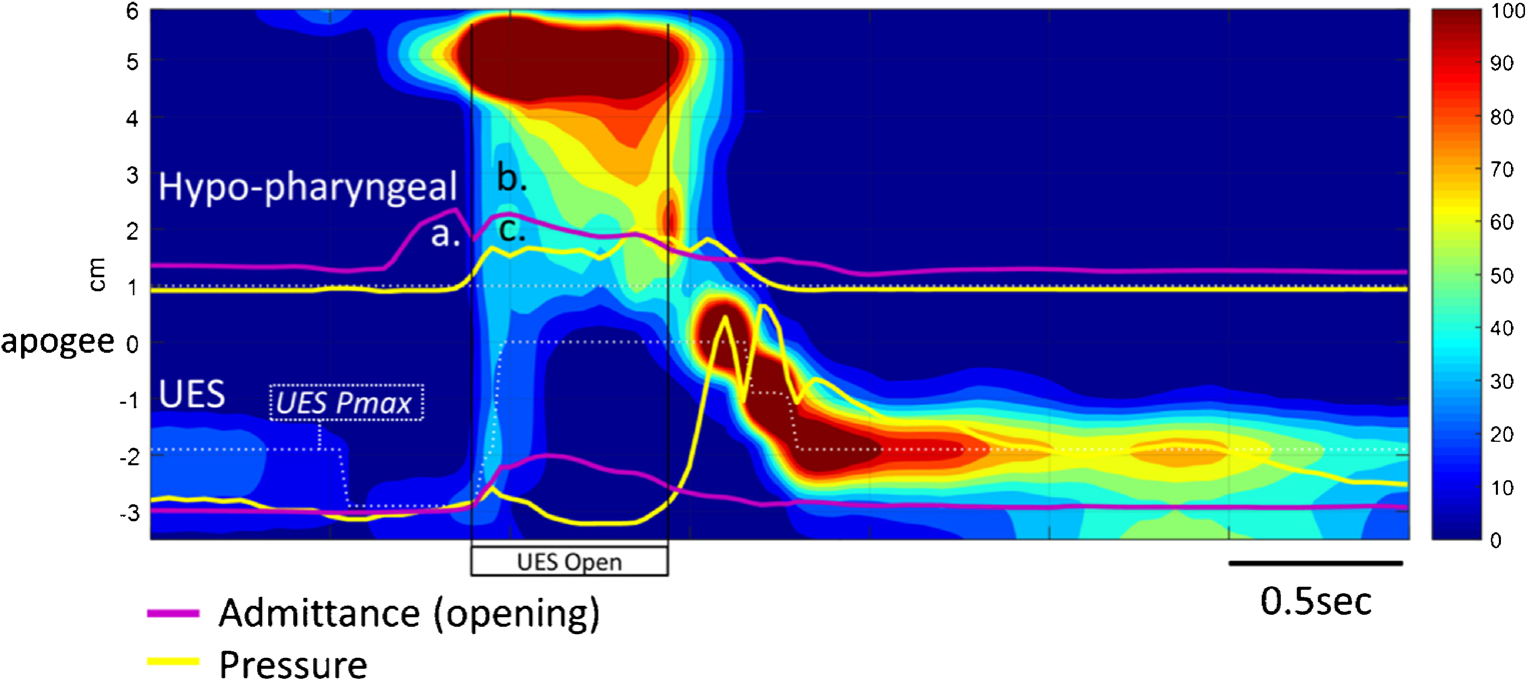
Example case 4 (C4 in Fig. 9) from a 70-year-old female patient with motor neurone disease (MND). Qualitatively, pharyngeal pressure topography suggests that pre-deglutative UES basal pressures were disproportionately low when compared to the good pressure generation/contractility of the pharynx and UES (post-deglutitive). Hypo-pharyngeal admittance shows clear separation of the lingual (*a*.) and pharyngeal (*b*.) phase of propulsion. However, in contrast to case 1, phase 1 precedes UES opening. As with case 3, hypo-pharyngeal admittance (*b*.) and pressure (*c*.) rise together (passive distension), a pattern consistent with flow resistance at the level of the UES rather than below. Absence of other structural pathology on radiology suggests that this is consistent with UES dysfunction of a neuro-myogenic origin

**Fig. 8:**
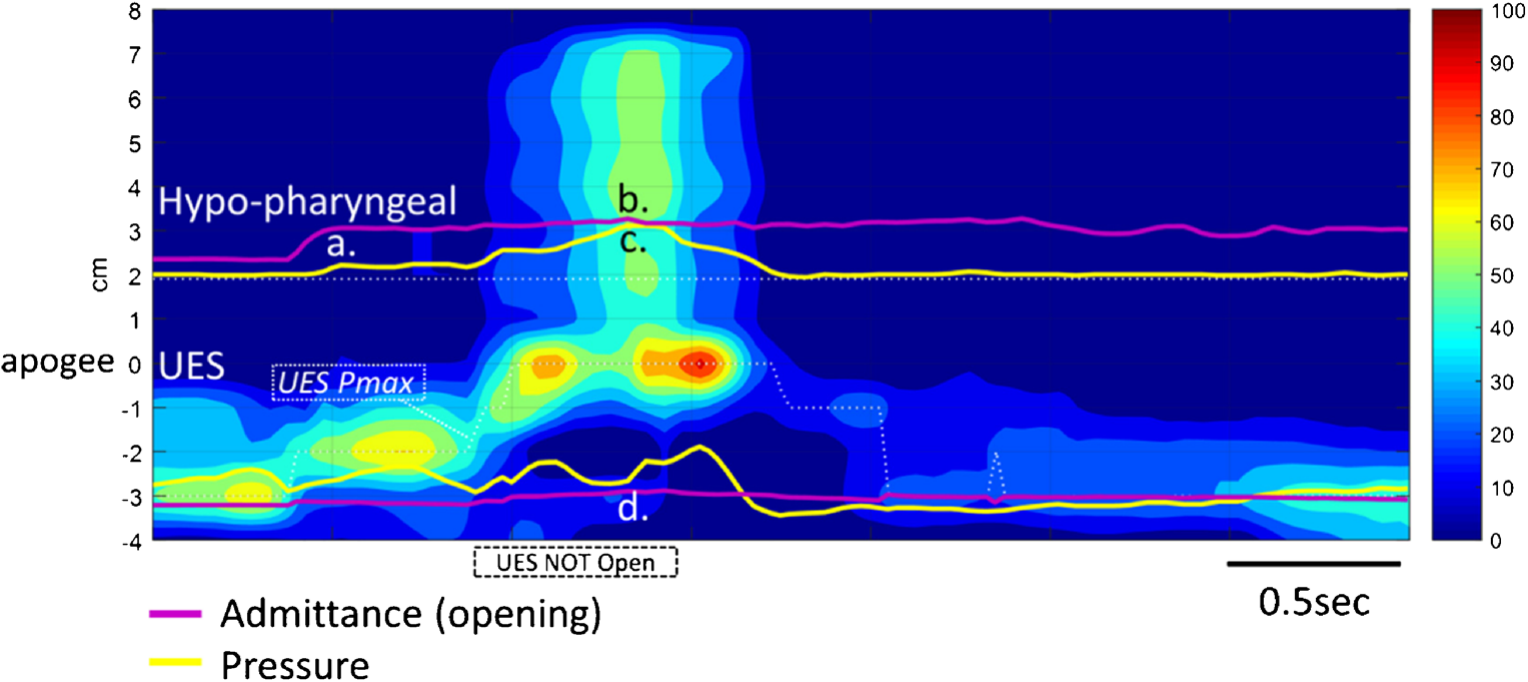
Example case 5 (C5 in Fig. 9) from a 70-year-old male patient who suffered a brainstem stroke. Qualitatively, this is a highly abnormal swallow. Hypo-pharyngeal admittance shows bolus arriving within the pharynx well ahead any attempt to swallow (*a*.). The pharynx remains dilated with bolus for the duration of this tracing. The hypo-pharyngeal admittance remains elevated (*b*.) and does not change when an attempt to swallow causes “pan-pressurisation” of the pharyngeal chamber (*c*.). The UES does not relax during the attempt swallow and UES admittance does not increase (*d*.), consistent with UES not opening and the pharyngeal chamber failing to empty

**Fig. 9:**
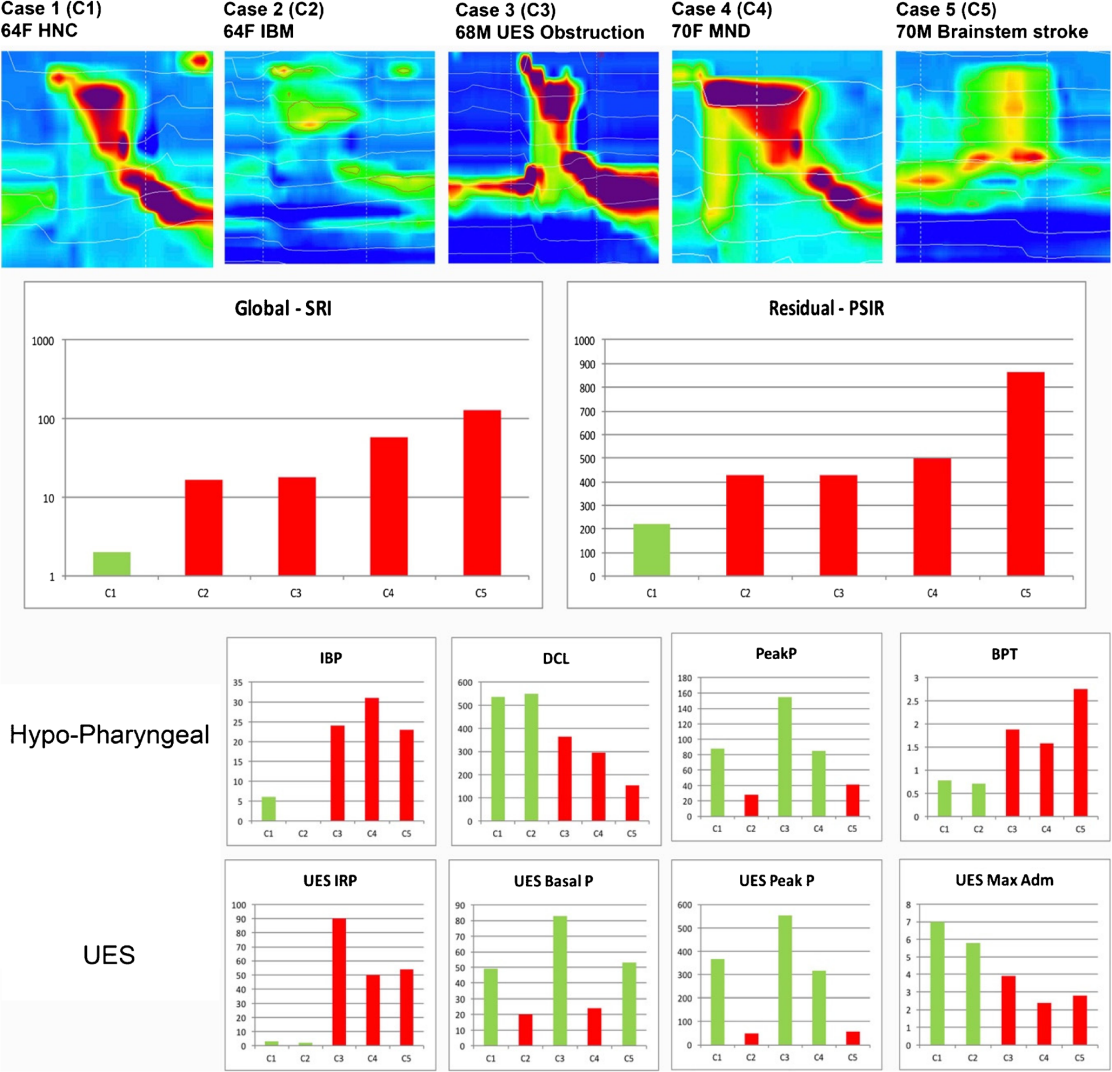
Mean swallow function variables for patient cases. Data are based on 5 × 10 ml saline bolus swallows; *red columns* indicate swallow function variable lies outside the normal range. *C1*, post head and neck cancer radiochemotherapy; *C2*, inclusion body myositis; *C3*, UES obstructive pathology; *C4*, motor neurone disease; *C5*, brainstem stroke

We have assembled the results from five dysphagia patients who underwent pharyngeal HRIM in our laboratory. Written informed consent was obtained from all subjects in order to publish de-identified case details (Southern Adelaide Clinical Human Research Ethics, Protocol 11/283). For each case we present an example 10 ml swallow with associated commentary based upon (i) qualitative review of a single representative swallow (Figs. 4, 5, 6, 7 and 8) and (ii) quantitative data for swallow function variables, for ease of interpretation these data are based on the averages of 5 × 10 ml saline swallows only (Fig. 9).

### Case 1 (C1) 64F following surgery and chemoradiotherapy (12 months) for T2N1M0 sarcomatoid oropharyngeal cancer. Ongoing dysphagia and coughing with solid bolus

Qualitative assessment (Fig. 4) suggests that pressure generation is intact. Hypo-pharyngeal admittance shows some evidence of pre-swallow bolus presence and separation of the lingual and pharyngeal phases of propulsion. Hypo-pharyngeal distension pressures are low during lingual propulsion (phase 1) but increase during pharyngeal propulsion (phase 2). UES pressures show complete UES relaxation. The pattern of distension pressure increase, which is absent during lingual propulsion and emerges during pharyngeal propulsion, is evidence for luminal restriction inferior to the UES.

Quantitative assessment (Fig. 9) showed that all swallow function variables were within normal limits. These findings are in keeping with radiology, which showed occasional premature spillage to the hypopharynx, no aspiration (penetration-aspiration scale (PAS) = 2; Material enters the airway, remains above the vocal folds, and is ejected from the airway) and only minor levels of post-swallow residue. The measured degree of flow resistance was low (normal IBP) suggesting only minor obstructive pathology. Radiology showed no-obvious stricture. However, as the pattern of intra-bolus pressurisation described above cannot be considered normal (i.e. not seen in healthy subjects), this situation should be monitored and a repeat investigation performed should symptoms worsen.

### Case 2 (C1): 64F subject with a 6-month history of proximal limb muscle weakness and progressive dysphagia, diagnosed as having inclusion body myositis (IBM)

Qualitative assessment (Fig. 5) suggests that pressure generation was globally weak, with a notable absence of hypo-pharyngeal constrictor activity and no visible hypo-pharyngeal distension pressures. Hypo-pharyngeal admittance shows no evidence of pre-swallow bolus presence. There is no separation into the lingual and pharyngeal phases of propulsion. However, in this patient with weak/absent pharyngeal activity, the single admittance peak most likely represents lingual propulsion only.

Quantitative assessment (Fig. 9) showed an abnormal SRI, consistent with aspiration risk, and abnormal PSIR, consistent with significant post-swallow residual. These global findings are in keeping with radiology, which shows significant post-swallow residue in both the valleculae and piriform sinuses, leading to post-swallow aspiration (PAS 5; Material enters the airway, contacts the vocal folds, and is not ejected from the airway).

The specific pressure variables confirmed profound weakness of the hypo-pharyngeal constrictor’s and criocopharyngeus muscle activity. Both pre- and post-deglutitive UES contractility were abnormal. Measures of intra-bolus pressure, UES opening and flow timing were all normal, consistent with good lingual bolus propulsion and sufficient UES opening. Note: In circumstances of absent, “non-propulsive”, hypo-pharyngeal contractility, low IBP reading do not necessarily exclude reduced UES opening and the literature suggests that cases of inflammatory myopathy are often associated restricted sphincter opening [20]. However normal UES admittance values confirm that UES opening is adequate in this case. These findings suggest that lingual and suprahyoid muscle function are less involved at this time (moderate pressure generation can be seen at the tongue base on the qualitative assessment).

### Case 3 (C2) 68M who presented with longstanding oropharyngeal dysphagia (coughing, choking when swallowing textured foods)

Qualitative assessment (Fig. 6) suggests that pressure generation is intact. Hypo-pharyngeal admittance shows no evidence of pre-swallow bolus presence. There is no clear separation of the lingual and pharyngeal phases of propulsion and hypo-pharyngeal distension pressures increase immediately with the onset of lingual propulsion suggesting flow resistance at the level of the UES (in contrast to case 1, who exhibits evidence of flow resistance below the level UES). UES pressures show incomplete UES relaxation.

Quantitative assessment (Fig. 9) showed an abnormal SRI, consistent with aspiration risk, and abnormal PSIR and BPT, consistent with significant post-swallow residual. UES maximum admittance was abnormal consistent with restricted UES opening. Of specific swallow function variables, findings of a high IRP, high IBP and short DCL are consistent with abnormal flow resistance at the UES. Contractility of the hypopharynx and UES was normal. These findings are in keeping with radiology showing obstructive pathology. Most notably an impression occluding >50% of the lumen at the level of UES. Prominent cervical osteophytes were also noted below the level of UES. The penetration-aspiration scale on radiology revealed laryngeal penetration (PAS = 5; Material enters the airway, contacts the vocal folds, and is not ejected from the airway).

### Case 4 (C4) 70F with bulbar onset motor neurone disease (MND). Clinically prolonged eating duration due to a need for smaller mouthfuls to avoid choking. A percutaneous endoscopic gastrostomy (PEG) was placed for supplemental feeding and hydration

Qualitative assessment (Fig. 7) suggests that pre-deglutitive UES basal pressures were disproportionately lower than UES contractile pressures. Hypo-pharyngeal admittance shows bolus presence prior to UES opening. There is clear separation of the lingual and pharyngeal phases of propulsion. However, in contrast to case 1, phase 1 is mistimed and precedes UES opening. Hypo-pharyngeal distension pressures indicate flow resistance at the level of the UES.

Quantitative assessment (Fig. 9) showed an abnormal SRI, consistent with aspiration risk, and abnormal PSIR and BPT, consistent with significant post-swallow residual. UES maximum admittance was abnormal, consistent with restricted UES opening. Of specific swallow function variables, findings of a high IRP, high IBP and short DCL are consistent with abnormal flow resistance at the UES. Pre-deglutitive UES basal tone was weak in contrast to contractility of the hypopharynx and UES which were normal.

These findings are consistent with UES dysfunction due to combined neuro-myogenic and structural changes, namely neural de-innervation leading to weak contractility and muscle inactivity in turn leading to loss of muscle compliance. Suprahyoid muscle involvement results in failure of the extrinsic UES opening mechanism. Consequently, the UES must be pushed open by the intra-bolus pressure generated by luminal closure of the pharyngeal chamber above. CP muscle involvement results in low tone and loss of compliance meaning that the pharynx must work even harder to achieve trans-sphinteric flow. Marked decompensation of the swallow can be anticipated if pharyngeal contractility, currently least affected, weakens as a consequence of MND progression.

Radiology revealed difficulty with oral bolus control, with subsequent premature bolus spillage. There was significant pharyngeal residue, when swallowing bolus of increased consistency. Cricopharyngeal bar inferior to the UES was evident; however, its location suggests that it is not obstructive to bolus flow. There was some evidence of laryngeal penetration during liquid swallows (PAS = 3; Material enters the airway, remains above the vocal folds) and marked residue retention during semi-solids.

### Case 5 (C5) 70M 6 weeks after a brainstem stroke. Profoundly dysphagic and dysphonic. PEG fed and effectively nil by mouth other than small volumes of thickened fluids. Study performed carefully with constant physician supervision and instructed throat clearing after each bolus

Qualitative assessment (Fig. 8) shows a highly abnormal swallow. Hypo-pharyngeal admittance shows bolus arriving within the pharynx well ahead of any attempt to swallow. The pharynx remains dilated with bolus for the duration of this tracing. An attempt to swallow causes pan-pressurisation of the pharyngeal chamber. However, the hypo-pharyngeal admittance remains elevated indicating that the lumen does not close. The UES does not relax during the attempt swallow, consistent with a persistent, neurogenically driven muscle tone (as previously described in neurogenic dysphagia [9]), and UES admittance does not increase, consistent with UES not opening and the pharyngeal chamber failing to empty.

Quantitative assessment (Fig. 9) showed an extreme SRI, consistent with aspiration risk, and abnormal PSIR and BPT, consistent with significant post-swallow residual. UES maximum admittance was abnormal, consistent with reduced UES opening. Every swallow function variable, with the exception of pre-deglutitive UES basal tone, was highly abnormal in this case.

These findings are consistent with total failure of pharyngeal swallow.

These findings are in keeping with radiology showing premature spillage of the bolus due to poor oro-lingual control, non-opening of the UES, with consequent retention of bolus in the hypopharynx and overt aspiration (PAS = 8; Material enters the airway, passes below the vocal folds, and no effort is made to eject).

The patient had a major ischemic brainstem stroke affecting several aspects of his oropharyngeal swallow. Despite mechanical dilatation and botulinum toxin injection of the UES, he remains dysphagic after 6 months related to incoordination of the swallow response and profound pharyngeal weakness. The extreme level SRI reinforces the catastrophic, multi-factorial, nature of the disorder and therefore likely failure of therapies that target the UES only.

## Conclusion

Pharyngeal manometry may have come of age. Solid-state high-resolution manometry and the addition of impedance (HRIM) overcomes many technical challenges associated with recording reliably and meaningfully from the pharyngeal region and offers the potential for a biomechanically based “value adding” assessment of swallowing to become clinical routine.

In the global SRI, PSIR and swallow function variables, we have validated a range of objective measures that can detect swallowing dysfunction. These can be tracked over time in order to measure the efficacy and durability of swallowing interventions. In order to progress this field, the next challenge will be to use this biomechanically based approach to study the effects of swallowing interventions, which are commonly used in clinical practice despite a paucity of evidence.

## References

[1] Omari TI, Jones CA, Hammer MJ, Cock C, Dinning P, Wiklendt L (2016). Predicting the activation states of the muscle governing upper esophageal sphincter relaxation and opening. Am J Physiol Gastrointest Liver Physiol.

[2] • Cock C, Jones CA, Hammer MJ, Omari TI, McCulloch TM. Modulation of upper esophageal sphincter (UES) relaxation and opening during volume swallowing. Dysphagia. 2016. doi 10.1007/s00455-016-9744-4. Sensory modulation of swallowing assessed by pressure-flow analysis. **Mechanism of UES opening shifts from neuromyogenic to bolus based distention with volume increase. Optimal biomechanical opening at 10-15ml in young healthy volunteers**. 10.1007/s00455-016-9744-4 PMC6442470 27534548

[3] Cook IJ, Dodds WJ, Dantas RO, Kern MK, Massey BT, Shaker R (1989). Timing of videofluoroscopic, manometric events, and bolus transit during the oral and pharyngeal phases of swallowing. Dysphagia.

[4] Cook IJ, Dodds WJ, Dantas RO, Massey B, Kern MK, Lang IM (1989). Opening mechanisms of the human upper esophageal sphincter. Am J Physiol.

[5] Omari TI, Dejaeger E, Tack J, Van Beckevoort D, Rommel N (2013). Effect of bolus volume and viscosity on pharyngeal automated impedance manometry variables derived for broad dysphagia patients. Dysphagia.

[6] Rosenbek JC, Robbins JA, Roecker EB, Coyle JL, Wood JL (1996). A penetration-aspiration scale. Dysphagia.

[7] Molfenter SM, Steele CM (2013). The relationship between residue and aspiration on the subsequent swallow: an application of the normalized residue ratio scale. Dysphagia.

[8] Pal A, Williams RB, Cook IJ, Brasseur JG (2003). Intrabolus pressure gradient identifies pathological constriction in the upper esophageal sphincter during flow. Am J Physiol Gastrointest Liver Physiol.

[9] Williams RB, Wallace KL, Ali GN, Cook IJ (2002). Biomechanics of failed deglutitive upper esophageal sphincter relaxation in neurogenic dysphagia. Am J Physiol Gastrointest Liver Physiol.

[10] Olsson R, Nilsson H, Ekberg O (1994). Simultaneous videoradiography and computerized pharyngeal manometry – videomanometry. Acta Radiol.

[11] Nativ-Zelzer N, Kahrilas PJ, Logemann JA (2012). Manofluorography in the evaluation of oropharyngeal dysphagia. Dysphagia.

[12] Omari TI, Rommel N, Szczesniak MM, Fuentealba S, Dinning PG, Davidson GP (2006). Assessment of intraluminal impedance for the detection of pharyngeal bolus flow during swallowing in healthy adults. Am J Physiol Gastrointest Liver Physiol.

[13] Szczesniak MM, Rommel N, Dinning PG, Fuentealba S, Cook IJ, Omari TI (2008). Optimal criteria for detecting bolus passage across the pharyngo-esophageal segment during the normal swallow using intraluminal impedance recording. Neurogastroenterol Motil.

[14] Omari TI, Papathanasopoulos A, Dejaeger E, Wauters L, Scarpellini E, Vos R (2011). Reproducibility and agreement of pharyngeal automated impedance manometry with videofluoroscopy. Clin Gastroenterol Hepatol.

[15] • Omari TI, Savilampi J, Kokkinn K, Schar M, Lamvik K, Doeltgen S, et al. The reliability of pharyngeal high resolution manometry with impedance for derivation of measures of swallowing function in healthy volunteers. Int J Otolaryngol. 2016;2016:2718482. doi:10.1155/2016/2718482. **Recent paper assessing inter-, intra- and test-retest reliability of pharyngeal manometry using pressure flow analyses. This study demonstrated a high degree of reliability for intra-bolus pressure and timing measurements, including of test -retest, but less reliable test-retest results for contractile pressure-based metrics**. 10.1155/2016/2718482 PMC4848412 27190520

[16] •• Cock C, Besanko L, Kritas S, Burgstad CM, Thompson A, Heddle R, et al. Maximum upper esophageal sphincter (UES) admittance: a non-specific marker of UES dysfunction. Neurogastroenterol Motil. 2016;28:225–33. **Paper describing Max UES Adm as a marker for UES dysfunction in older subjects, subjects with motor neurone disease and subjects with cricopharyngeal bar, which were often shown to be non-obstructive in nature**. 10.1111/nmo.12714 26547361

[17] Omari TI, Ferris L, Dejaeger E, Tack J, Van Beckevoort D, Rommel N (2012). Upper esophageal sphincter impedance as a marker of sphincter opening diameter. Am J Physiol Gastrointest Liver Physiol.

[18] Zhang T, Szczesniak M, Maclean J, Betrand P, Wu PI, Omari T (2016). Biomechanics of pharyngeal deglutitive function following total laryngectomy. Otolanrygol Head Neck Surg.

[19] Cook IJ, Gabb M, Panagopoulos V, Jamieson GG, Dodds WJ, Dent J (1992). Pharyngeal (Zenker’s) diverticulum is a disorder of upper esophageal sphincter opening. Gastroenterology.

[20] Williams RB, Grehan MJ, Hersch M, Andre J, Cook IJ (2003). Biomechanics, diagnosis, and treatment outcome in inflammatory myopathy presenting as oropharyngeal dysphagia. Gut.

[21] • Doeltgen S, Omari TI, Savilampi J. Remifentanil alters sensory neuromodulation of swallowing in healthy volunteers: quantification by a novel pressure-impedance analysis. Am J Physiol Gastrointest Liver Physiol. 2016;310:G1176–82. **Paper describing a reduction in the duration of UES relaxation following administration of the mu-opioid receptor agonist, remifentanil**. 10.1152/ajpgi.00138.2016 27151943

[22] Ferris L, Omari T, Selleslach M, Dejaeger E, Tack J, Vanbeckevoort D (2015). Pressure flow analysis in the assessment of preswallow pharyngeal bolus presence in dysphagia. Int J Otolaryngol.

[23] •• Omari TI, Dejaeger E, Van Beckevoort D, Goeleven A, Davidson GP, Dent J, et al. A method to objectively assess swallow function in adults with suspected aspiration. Gastroenterology. 2011;140:1454–63. **Description of the swallow risk index during pressure-flow analysis, as a composite measure of global swallowing dysfunction and aspiration risk**. 10.1053/j.gastro.2011.02.051 21354152

[24] Omari TI, Dejaeger E, Tack J, Van Beckevoort D, Rommel N (2012). An impedance-manometry based method for non-radiological detection of pharyngeal post-swallow residue. Neurogastroenterol Motil.

[25] Weijenborg PW, Kessing BF, Smout AJPM, Bredenoord AJ (2014). Normal values for solid state esophageal high-resolution manometry in a European population: an overview of all current metrics. Neurogastroenterol Motil.

[26] Nativ-Zelzer N, Logemann JA, Zecker SG, Kahrilas PJ (2016). Pressure topography metrics for high-resolution pharyngeal-esophageal manofluorography—a normative study of younger and older adults. Neurogastroenterol Motil.

[27] Lee TH, Lee JS, Park JW, Cho SJ, Hong SJ, Jeon SR (2014). High resolution impedance manometry facilitates assessment of pharyngeal residue and oropharyngeal dysphagic mechanisms. Dis Esophagus.

[28] Savilampi J, Omari T, Magnuson A, Ahlstrand R (2016). Effects of remifentanil on pharyngeal swallowing. Eur J Anaesthesiol.

